# Neutrophil extracellular traps in cardiac hypertrophy: a KLF2 perspective

**DOI:** 10.1172/JCI156453

**Published:** 2022-02-01

**Authors:** Dario F. Riascos-Bernal, Nicholas E.S. Sibinga

**Affiliations:** 1 Department of Medicine (Cardiology) and Wilf Family Cardiovascular Research Institute and; 2 Department of Developmental and Molecular Biology, Albert Einstein College of Medicine, Bronx, New York, USA.

## Abstract

About 6 million adults in the United States have heart failure, and the mortality five years after diagnosis remains high at approximately 50%. Incomplete understanding of disease pathogenesis limits therapeutics, especially in the case of heart failure with preserved ejection fraction, a condition commonly associated with cardiac hypertrophy. Neutrophils, the most abundant leukocyte in blood, have functions beyond antimicrobial activity and participate in both sterile inflammation and disease; however, their role in nonischemic cardiac hypertrophy and heart failure is underexplored. In this issue of the *JCI*, Tang et al. show that neutrophil extracellular trap (NET) formation contributes to cardiac hypertrophy and dysfunction in a mouse model of angiotensin II–induced cardiomyopathy, and that Krüppel-like factor 2 (KLF2) functions in neutrophils to oppose this process. Whether a neutrophil-centered strategy may benefit patients with cardiac hypertrophy and failure deserves further investigation.

## Neutrophils in cardiac hypertrophy

Cardiac hypertrophy is characterized by enlarged cardiomyocyte size and mass, typically in response to increased hemodynamic load. It can be an adaptive response, referred to as physiological cardiac hypertrophy, as observed during pregnancy or in response to endurance exercise. But it can also be a detrimental response called pathological cardiac hypertrophy, as seen in patients with chronic arterial hypertension, valvulopathies, myocardial infarction, or those bearing mutations in sarcomeric proteins (1). Pathological cardiac hypertrophy may lead to heart failure, arrhythmias, or death (1). About 6 million adults in the United States suffer from heart failure, and the prevalence of this condition is increasing, particularly in older age groups (2, 3). Moreover, mortality in patients with heart failure five years after diagnosis remains high at approximately 50% (2, 3), and the incidence of heart failure with preserved ejection fraction, which is commonly associated with left ventricular hypertrophy (4), is increasing (2). Heart failure is thus a major burden on public health, and better understanding of the pathogenetic factors that drive pathological cardiac hypertrophy has both high importance and urgency.

Current mechanistic understanding of pathological cardiac hypertrophy is far from a blank slate. Along with increased protein synthesis and cardiomyocyte growth, this condition has been characterized by induction of fetal genes, altered calcium handling and sarcomere structure, mitochondrial dysfunction and cell metabolic reprogramming, impaired angiogenesis, and increased cell death and fibrosis (1). Several molecular contributors (e.g., angiotensin II, endothelin 1, catecholamines, mTOR signaling, natriuretic peptides, mechanosensors, sarcomeric proteins, epigenetic regulators, noncoding RNAs, cellular metabolic remodeling, and inflammatory signaling pathways) have been implicated in pathological cardiac hypertrophy (1, 5). Similarly, multiple cellular players (e.g., cardiomyocytes, endothelial cells, fibroblasts, and immune cells) participate in disease pathogenesis (5). Nevertheless, the understanding of cardiac hypertrophy and failure is incomplete, which limits therapeutic strategies and in turn perpetuates the large burden of disease. There are, for example, no specific and effective treatments for heart failure with preserved ejection fraction, a condition commonly characterized by left ventricular hypertrophy (2, 4). In this issue of the *JCI*, Tang et al. provide a translationally provocative contribution to the understanding of nonischemic cardiac hypertrophy and failure (6).

In comparison with studies of resident and infiltrating immune cells in cardiac remodeling during and after acute myocardial infarction, research addressing the contribution of immune cells to nonischemic cardiac hypertrophy is relatively recent and not fully developed. Some progress has been made in understanding the distinct roles of different subsets of macrophages and T cells, as well as the influence of mast cells, in cardiac hypertrophy (5). In contrast, there is a notable paucity of studies addressing the importance of neutrophils in this context. One recent study showed that neutrophil depletion reduces cardiac hypertrophy and dysfunction induced by pressure overload (i.e., transverse aortic constriction) in mice (7). Tang et al. provide evidence that neutrophils contribute to cardiac hypertrophy and dysfunction induced by angiotensin II (6).

Using a loss-of-function strategy, Tang et al. show that depletion of neutrophils with anti-Ly6G antibody reduces cardiac hypertrophy and dysfunction induced by angiotensin II infusion in mice (6). Conversely, acute neutrophilia achieved by neutrophil transfusion aggravates cardiac hypertrophy and dysfunction (6). Moreover, genetic deletion in the myeloid compartment (including monocytes/macrophages and neutrophils) of Krüppel-like factor 2 (KLF2), a transcription factor that has been shown to oppose inflammation (8), increases neutrophil accumulation in the myocardium and enhances cardiac hypertrophy, fibrosis, and dysfunction upon angiotensin II infusion; notably, this cardiac phenotype was also suppressed by depleting neutrophils with anti-Ly6G antibody (6). Finally, deletion of KLF2 only in monocytes/macrophages did not increase angiotensin II–induced cardiac hypertrophy and dysfunction (6). Altogether, these in vivo studies suggest that, at least in the context of angiotensin II–induced cardiac hypertrophy and failure, loss of KLF2 in neutrophils leads to exacerbated cardiac neutrophil accumulation that in turn promotes cardiac hypertrophy and dysfunction.

Interestingly, Tang et al. also studied peripheral blood leukocytes from a cohort of patients with heart failure and age-matched controls, and show that mRNA levels of *KLF2* were reduced in total leukocytes and neutrophils of patients with heart failure (6). Although the small sample size prevents generalization of the inverse association between *KLF2* expression and heart failure, it is nevertheless an intriguing observation that requires further investigation. Whether the detrimental effect on cardiac function caused by loss of KLF2 expression in mouse neutrophils translates to human heart failure pathophysiology is an open and relevant question, the answer to which could support a therapeutic strategy aimed at promoting KLF2 function in neutrophils in patients with cardiac hypertrophy and failure. Will a therapeutic approach that targets neutrophil function be available in the future to slow or even reverse heart failure with preserved ejection fraction? Such an accomplishment would certainly be a boon to patients plagued by this relatively common condition.

## Cardiac hypertrophy features neutrophil extracellular trap formation

Neutrophils are the most abundant leukocytes in blood and are classically recognized for their antimicrobial function. In addition, they are also important contributors to sterile inflammation and to diseases that feature an inflammatory component such as atherosclerosis or the response to acute myocardial infarction (9, 10). It was initially thought that neutrophils mediate their antimicrobial functions mainly by phagocytosis and degranulation; however, evidence that these cells also release neutrophil extracellular traps (NETs) has emerged since the early 2000s (11, 12). NETs are web-like structures made of a scaffold of loose chromatin (DNA and histone proteins) coated with elastase, myeloperoxidase, and cathepsin G, among other proteins. NETs are released to the extracellular compartment from neutrophils by either a lytic or nonlytic mechanism (11, 12). Although NETs contribute to clearance of viral, bacterial, fungal, and parasitic infections, excessive or persistent NET formation may cause tissue damage and contribute to disease progression (11, 12). Therefore, regulation of NET formation and release should be tightly regulated to favor beneficial effects and avoid detrimental consequences. Although molecular understanding of NET formation has improved (11, 12), mechanisms that regulate NET formation are not fully elucidated and are likely to be complex and context dependent.

The studies of Tang et al. show that NET formation plays a pathogenic role in angiotensin II–induced cardiac hypertrophy in mice, and that loss of KLF2 function in neutrophils favors NET formation in the heart (6). Notably, disrupting NET formation by treating mice with DNase I decreased the deleterious effect of neutrophilia in this setting (6). On the other hand, mice lacking expression of KLF2 in neutrophils and macrophages exhibited increased neutrophil accumulation, NET formation, and cardiac hypertrophy. Once more, decreasing NET formation in these mice by either DNase I treatment or pharmacological inhibition of peptidyl arginine deiminase 4 reduced cardiac hypertrophy, fibrosis, and dysfunction (6). Altogether, these in vivo studies support the idea that NETs contribute to the development of cardiac hypertrophy induced by angiotensin II, and that KLF2 opposes NET formation. Consistently, in vitro studies showed that KLF2-deficient neutrophils exhibited enhanced NET formation (6). Tang et al. also found that, in addition to having lower expression of *KLF2* in neutrophils, patients with heart failure had higher serum levels of markers of NET formation (6). This observation suggests that KLF2 may also oppose NET formation in humans in the context of heart failure; however, additional clinical studies are necessary to confirm this possibility more broadly.

The inverse relationship between KLF2 function in neutrophils and NET formation (i.e., higher neutrophil KLF2 function and lower NET formation) proposed by Tang, et al. raises the question as to whether KLF2 determines the ability of neutrophils to form NETs. It has been shown that not all stimulated neutrophils release NETs (11). Do KLF2 expression levels distinguish neutrophil subsets that may differ in NET-forming capacity in humans?

## From NET formation to cardiac hypertrophy

**Figure 1:**
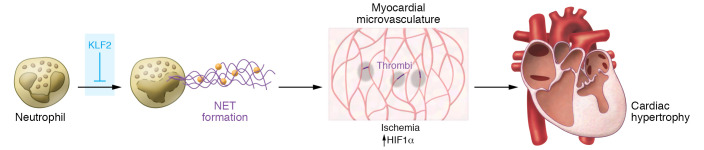
Neutrophil extracellular trap formation promotes cardiac hypertrophy and dysfunction. In a mouse model of angiotensin II–induced cardiac hypertrophy, neutrophils accumulate in the heart and release neutrophil extracellular traps (NETs), a process opposed by KLF2. NETs localize to the myocardial microvasculature and trigger thrombosis, vascular occlusion, reduction of blood flow, and localized myocardial ischemia, cell death, and capillary rarefaction. This process is also characterized by increased levels of HIF1α. These events result in cardiac hypertrophic remodeling.

How NET formation leads to cardiac hypertrophy has not been fully elucidated. Tang et al. observed that mice lacking KLF2 in the myeloid compartment showed increased neutrophil accumulation, NET formation, and cardiac hypertrophy upon angiotensin II treatment. Detailed analyses yielded several important clues as to pathogenetic mechanisms: (a) most neutrophils and NETs localized to small vessels wherein thrombi were also observed; (b) myocardial capillary density was reduced (i.e., capillary rarefaction); (c) myocardial blood flow was reduced and markers of ischemia were increased, including higher expression of hypoxia-inducible factor 1α (HIF1α); (d) treatment with heparin, an anticoagulant frequently used in patients (13), reduced NET formation, cardiac hypertrophy, and dysfunction; and (e) concomitant deletion of HIF1α also decreased NET formation, cardiac hypertrophy, and dysfunction (6). These observations support the idea that KLF2 loss results in increased NET formation and thrombosis in the microvasculature, which in turn leads to localized myocardial ischemia, cell death, and microvascular rarefaction (Figure 1), phenomena that have been associated with cardiac hypertrophy (1, 14–16).

Whether the protective role of KLF2 in neutrophils reported by Tang et al. (6) in mouse models of angiotensin II–induced cardiac hypertrophy also operates in heart failure in humans remains unknown. However, the findings of Tang et al. (6) provide a strong rationale to pursue studies that tackle this gap of knowledge. This avenue of research may lead to therapeutic strategies for heart failure aimed at promoting KLF2 function in neutrophils, or limiting neutrophil activation, NET formation, or microvascular thrombosis and dysfunction.

## Version 1 02/01/2022

Electronic publication
